# Bleeding risk with dabigatran, rivaroxaban, warfarin, and antiplatelet agent in Asians with non-valvular atrial fibrillation

**DOI:** 10.18632/oncotarget.22026

**Published:** 2017-10-24

**Authors:** Yi-Hsin Chan, Yung-Hsin Yeh, Hui-Tzu Tu, Chi-Tai Kuo, Shang-Hung Chang, Lung-Sheng Wu, Hsin-Fu Lee, Lai-Chu See

**Affiliations:** ^1^ The Cardiovascular Department, Chang-Gung Memorial Hospital, Taoyuan, Taiwan; ^2^ College of Medicine, Chang-Gung University, Taoyuan, Taiwan; ^3^ Microscopy Core Laboratory, Chang-Gung Memorial Hospital, Taoyuan, Taiwan; ^4^ Department of Public Health, College of Medicine, Chang-Gung University, Taoyuan, Taiwan; ^5^ Center for Big Data Analytics and Statistics, Chang-Gung Memorial Hospital, Taoyuan, Taiwan; ^6^ Biostatistics Core Laboratory, Molecular Medicine Research Center, Chang-Gung University, Taoyuan, Taiwan; ^7^ Division of Rheumatology, Allergy and Immunology, Department of Internal Medicine, Chang-Gung Memorial Hospital, Taoyuan, Taiwan

**Keywords:** atrial fibrillation, direct thrombin inhibitor, factor xa inhibitor, hemorrhage, warfarin

## Abstract

It is not understood if dabigatran or rivaroxaban are superior to antiplatelet agents (AA) for safety outcomes in Asians with non-valvular atrial fibrillation (NVAF). In this study we evaluated the bleeding risk of dabigatran, rivaroxaban, warfarin and AA in Asians with NVAF. This national retrospective cohort study analyzed 6,600, 3,167, 5,338 and 8,238 consecutive NVAF patients taking dabigatran, rivaroxaban, warfarin or AAs (including aspirin, clopidogrel or ticlopidine), respectively, from June 1, 2012 to December 31, 2013. Propensity-score weighting was used to balance covariates across study groups. Patients were followed until the first occurrence of any bleeding outcome or the end of the study. The CHA_2_DS_2_-VASc scores were 4.1±1.6, 4.1±1.6, 3.3±1.8 and 2.4±1.6 for the dabigatran, rivaroxaban, warfarin, and AA groups, respectively. There were 5,822 (88.2%) and 164 (5.2%) patients taking low dose dabigatran and rivaroxaban, respectively. Hazard ratios (95% confidence intervals) for dabigatran, rivaroxaban, or warfarin versus AA were: intracranial hemorrhage, 0.36 (0.23-0.57;*PP*=0.0037) and 1.34 (0.89-2.02;*P*=0.1664); gastrointestinal bleeding, 0.44 (0.32-0.59;*PP*=0.0189); and all hospitalized major bleeding, 0.41 (0.32-0.53;*PP*=0.0644) and 0.90 (0.70-1.16;*P*=0.4130) after adjustment. The risk reduction of all major bleeding for dabigatran versus AA persisted on subgroup analysis. In conclusion, we observed that dabiagtran was associated with a lower risk of all major bleeding in Asians with NVAF, whereas rivaroxaban had a similar risk of all major bleeding compared with antiplatelet agents after adjustment of comorbidities.

## INTRODUCTION

Although randomized trials have shown that warfarin significantly decreased the risk of thromboembolic events by 62% in patients with atrial fibrillation (AF), vitamin K antagonists (VKAs) remained underutilized in real world clinical practice. [1–3] There is a higher risk of intracranial hemorrhage (ICH) and other major bleeding events in Asians taking warfarin, compared to non-Asians. [4, 5] Data from a real-world registry study also showed that more than 65% of Asian patients on warfarin had a markedly low (16.7%) time in therapeutic range (TTR). [6] Aspirin reduces the risk of stroke in AF patients by about 20% and is commonly used in AF patients for whom warfarin therapy is unsuitable. [1] Although aspirin is not as efficacious as warfarin in reducing the thromboembolic risk, it may be a more convenient choice than warfarin in certain patient populations, and may therefore be prescribed as an alternative to warfarin for stroke prevention especially in Asia.

The AVEROOSES trial reported that apixaban was non-inferior to aspirin for the risk of major bleeding (1.4%/year versus 1.2%/year, respectively) in AF patients. [7] These data suggested that apixaban is an attractive alternative to aspirin for stroke prevention in AF patients unsuitable for warfarin. However, there are no studies currently which directly compared the safety outcomes in AF patients treated with other non-vitamin K antagonist oral anticoagulants (NOACs), (e.g. dabigatran, edoxaban or rivaroxaban) versus those treated with aspirin. It is also unclear whether dabigatran or rivaroxaban is superior to antiplatelet agents (AA) (including aspirin, clopidogrel, or ticlopidine) for safety outcomes, specifically in Asians with AF. Therefore, in this study we evaluated the bleeding risk associated with dabigatran, rivaroxaban, warfarin compared to AA therapy in Asians with non-valvular AF.

## RESULTS

### Participants

Patient demographics and medication use before and after propensity score weighting are described in Tables 1 to 3. This study enrolled a total of 6,600, 3,167, 5,338 and 8,238 consecutive patients taking dabigatran, rivaroxaban, warfarin, and AA respectively, from June, 2012 to December, 2013. Analysis of the AA group showed that a total of 7,181 (87.2%), 470 (5.7%), 252 (3.1%), and 335 (4.1%) patients took aspirin, clopidogrel, ticlopidine, and dual AAs (mostly aspirin plus clopidogrel, n=319, 3.9%), respectively, for stroke prevention. Noted no patients have shifted between different NOACs (e.g. from dabigatran to rivaroxaban, and vice versa) during their whole following period. However, a small number of patients (n=463, 4.74%) shifted between different dosages of the same NOAC. For the dabigatran group (n=6,600), 292 patients had dose alteration (4.52%): 153 altered from 150 to 110 mg; 139 altered from 110 to 150 mg. For the rivaroxaban group (n=3,167), 171 patients had dose alteration (5.40%): 11 and 19 altered from 20 to 15 and 10 mg, respectively; 16 and 44 altered from 15 to 20 and 10 mg respectively; 21 and 60 altered from 10 to 20 and 15 mg, respectively. For the AA group, no patients have shifted between different AAs or dose adjustment. Since rivaroxaban was only approved after February, 2013 in Taiwan, we selected 4,616 patients from the AA group with a first prescription of AA after February, 2013 for a head-to-head comparison with the rivaroxaban group (Table 2). Before propensity score weighting, dabigatran, rivaroxaban, and warfarin groups were older, had a higher CHA_2_DS_2_-VASc score (heart failure, hypertension, age 75 years or older, diabetes mellitus, previous stroke or transient ischemic attack (TIA), vascular disease, age 65 to 74 years, female gender), HAS-BLED score (hypertension, abnormal renal or liver function, stroke, bleeding history, labile INR, age 65 years or older, and antiplatelet drug or alcohol use), and a higher proportion of comorbidities compared to the AA group. The CHA_2_DS_2_-VASc scores were 4.1±1.6, 4.1±1.6, 3.3±1.8 and 2.4±1.6 for the dabigatran, rivaroxaban, warfarin, and AA groups, respectively.

**Table 1:** Baseline characteristics of non-valvular atrial fibrillation (AF) patients taking dabigatran (D) and antiplatelet agents (AA), before and after propensity score weighting

	Propensity score weighting					
	Before			After		
	D(n=6,600)	AA(n=8,238)	ASMD	D(n=6,600)	AA(n=8,238)	ASMD
Age, yrs	75±10	68±14	0.5379	75±10	77±9	0.1677
<65	13%	40%		13%	11%	
65-74	30%	23%		30%	25%	
75-84	42%	23%		42%	46%	
>85	15%	14%		15%	18%	
Male	58%	58%	0.0034	58%	55%	0.0658
CHA_2_DS_2_-VASc	4.06±1.57	2.38±1.61	1.0520	4.06±1.57	4.43±1.75	0.2252
HAS-BLED	3.07±1.13	2.42±1.01	0.5988	3.07±1.13	3.71±1.02	0.5967
Chronic liver disease	27%	11%	0.4139	27%	27%	0.0100
Chronic kidney disease	22%	9%	0.3514	22%	24%	0.0501
Congestive heart failure	15%	6%	0.2828	15%	20%	0.1530
Hypertension	86%	57%	0.6614	86%	88%	0.0581
Hyperlipidemia	51%	22%	0.6271	51%	51%	0.0092
Diabetes mellitus	40%	20%	0.4541	40%	46%	0.1082
Previous stroke	35%	6%	0.7585	35%	42%	0.1625
Previous TIA	5%	1%	0.2689	5%	8%	0.1208
Myocardial infarction	2%	2%	0.0071	2%	1%	0.0749
Gout	29%	11%	0.4693	29%	33%	0.0869
Peripheral artery disease	0%	0%	0.0302	0%	0%	0.0302
Malignancy	8%	7%	0.0406	8%	9%	0.0123
History of bleeding	2%	1%	0.0624	2%	4%	0.1338
Use of NSAIDs	24%	27%	0.0724	24%	21%	0.0686
Use of PPI	5%	5%	0.0125	5%	4%	0.0227
Use of ACEI/ARB	62%	40%	0.4413	62%	60%	0.0399
Use of amiodarone	17%	26%	0.2253	17%	14%	0.0841
Use of beta-blocker	50%	47%	0.0630	50%	52%	0.0290
Use of diltiazem/verapamil	20%	22%	0.0299	20%	19%	0.0338
Use of digoxin	26%	18%	0.1888	26%	24%	0.0461
Use of statin	28%	12%	0.4265	28%	26%	0.0405
PCI	4%	2%	0.1178	4%	2%	0.1527
CABG	1%	0%	0.0709	1%	0%	0.0796

ACEI = angiotensin-converting-enzyme inhibitor; AF = atrial fibrillation; ARB =angiotensin II receptor antagonists; ASMD = absolute standardized mean difference; CABG = coronary artery bypass graft; CHA_2_DS_2_-VASc = congestive heart failure, hypertension, age 75 years or older, diabetes mellitus, previous stroke/transient ischemic attack, vascular disease, age 65 to 74 years, female; HAS-BLED = hypertension, abnormal renal or liver function, stroke, bleeding history, labile INR, age 65 years or older, and antiplatelet drug or alcohol use. Labile INR could not be determined from claims and was excluded from our scoring; NSAIDs = non-steroid anti-inflammatory drugs; PCI = percutaneous coronary intervention; PPI = proton pump inhibitor; TIA = transient ischemic attack

**Table 2:** Baseline characteristics of non-valvular atrial fibrillation (AF) patients taking rivaroxaban (R) and antiplatelet agents (AA), before and after propensity score weighting

	Propensity score weighting					
	Before			After		
	R(n=3,167)	AA(n=4,616)	ASMD	R(n=3,167)	AA(n=4,616)	ASMD
Age, yrs	76±9	68±14	0.6263	76±9	77±8	0.1064
<65	11%	42%		11%	10%	
65-74	30%	22%		30%	25%	
75-84	42%	22%		42%	48%	
>85	17%	14%		17%	17%	
Male	53%	58%	0.1069	53%	51%	0.0456
CHA_2_DS_2_-VASc	4.07±1.61	2.37±1.63	1.0551	4.07±1.61	4.34±1.57	0.1663
HAS-BLED	3.06±1.14	2.41±1.02	0.6040	3.06±1.14	3.70±1.00	0.6017
Chronic liver disease	27%	11%	0.4388	27%	30%	0.0633
Chronic kidney disease	21%	9%	0.3393	21%	23%	0.0316
Congestive heart failure	15%	6%	0.3054	15%	19%	0.0926
Hypertension	86%	57%	0.6702	86%	87%	0.0415
Hyperlipidemia	50%	22%	0.6162	50%	53%	0.0529
Diabetes mellitus	39%	20%	0.4233	39%	43%	0.0880
Previous stroke	30%	6%	0.6417	30%	37%	0.1550
Previous TIA	4%	1%	0.2298	4%	6%	0.0881
Myocardial infarction	3%	2%	0.0553	3%	1%	0.0997
Gout	28%	10%	0.4720	28%	32%	0.1041
Peripheral artery disease	0%	0%	0.0000	0%	0%	0.0000
Malignancy	9%	7%	0.0560	9%	8%	0.0137
History of bleeding	2%	1%	0.0868	2%	5%	0.1556
Use of NSAIDs	22%	27%	0.1239	22%	20%	0.0628
Use of PPI	7%	5%	0.0540	7%	7%	0.0025
Use of ACEI/ARB	60%	40%	0.4015	60%	59%	0.0331
Use of amiodarone	16%	26%	0.2500	16%	13%	0.0663
Use of beta-blocker	52%	48%	0.0882	52%	53%	0.0049
Use of diltiazem/verapamil	21%	21%	0.0104	21%	22%	0.0197
Use of digoxin	26%	18%	0.2009	26%	26%	0.0100
Use of statin	28%	12%	0.4031	28%	25%	0.0635
PCI	5%	2%	0.1785	5%	2%	0.1864
CABG	1%	0%	0.0827	1%	0%	0.0823

The abbreviations as in Table 1.

**Table 3:** Baseline characteristics of non-valvular atrial fibrillation (AF) patients taking warfarin (W) and antiplatelet agents (AA), before and after propensity score weighting

	Propensity score weighting					
	Before			After		
	W(n=5,338)	AA(n=8,238)	ASMD	W(n=5,338)	AA(n=8,238)	ASMD
Age, yrs	71±12	68±14	0.1809	71±12	70±10	0.0292
<65	31%	40%		31%	32%	
65-74	27%	23%		27%	26%	
75-84	29%	23%		29%	29%	
>85	13%	14%		13%	13%	
Male	55%	58%	0.0720	55%	53%	0.0231
CHA_2_DS_2_-VASc	3.25±1.80	2.38±1.61	0.5088	3.25±1.80	3.26±1.49	0.0051
HAS-BLED	2.61±1.33	2.42±1.01	0.1538	2.61±1.33	3.06±0.94	0.3927
Chronic liver disease	22%	11%	0.3004	22%	22%	0.0020
Chronic kidney disease	19%	9%	0.2913	19%	20%	0.0165
Congestive heart failure	14%	6%	0.2256	14%	14%	0.0069
Hypertension	74%	57%	0.3546	74%	74%	0.0018
Hyperlipidemia	40%	22%	0.3972	40%	40%	0.0116
Diabetes mellitus	33%	20%	0.3007	33%	33%	0.0051
Previous stroke	20%	6%	0.4364	20%	22%	0.0313
Previous TIA	2%	1%	0.1370	2%	3%	0.0102
Myocardial infarction	1%	2%	0.0530	1%	1%	0.0417
Gout	22%	11%	0.2986	22%	22%	0.0098
Peripheral artery disease	0%	0%	0.0000	0%	0%	0.0000
Malignancy	8%	7%	0.0207	8%	8%	0.0082
History of bleeding	2%	1%	0.0730	2%	3%	0.0168
Use of NSAIDs	27%	27%	0.0159	27%	26%	0.0075
Use of PPI	7%	5%	0.0828	7%	8%	0.0168
Use of ACEI/ARB	56%	40%	0.3248	56%	57%	0.0139
Use of amiodarone	29%	26%	0.0721	29%	28%	0.0318
Use of beta-blocker	55%	47%	0.1670	55%	56%	0.0037
Use of diltiazem/verapamil	23%	22%	0.0305	23%	22%	0.0232
Use of digoxin	27%	18%	0.2201	27%	27%	0.0105
Use of statin	20%	12%	0.2359	20%	19%	0.0177
PCI	2%	2%	0.0010	2%	1%	0.0674
CABG	0%	0%	0.0620	0%	0%	0.0360

The abbreviations as in Table 1.

### Incidence of bleeding outcome

**Figure 1:**
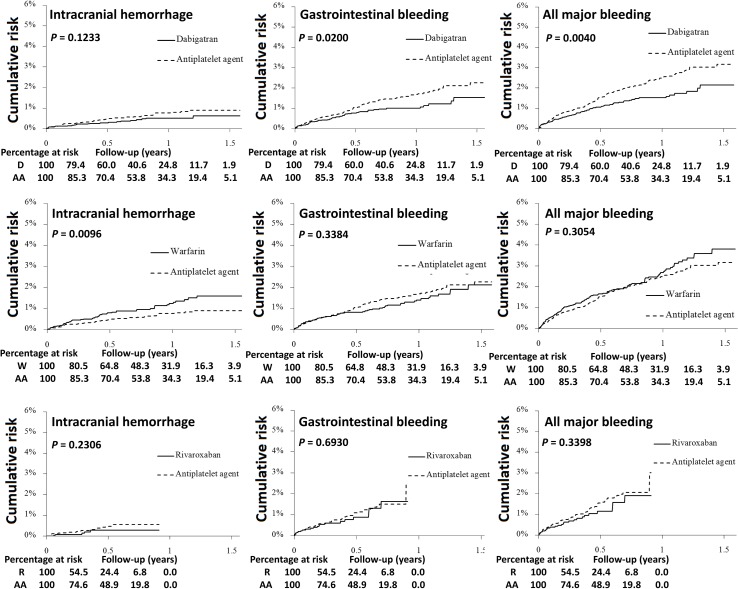
Cumulative risk of intracranial hemorrhage, gastrointestinal bleeding, and all major bleeding for non-valvular AF (NVAF) patients treated with dabigatran, warfarin or rivaroxaban versus AA before propensity score weighting Patients on dabigatran (solid line) have a lower risk of gastrointestinal bleeding and all major bleeding than those on antiplatelet agents (dotted line). Patients on warfarin (solid line) have a higher risk of intracranial hemorrhage than those on antiplatelet agents (dotted line). Patients on rivaroxaban (solid line) have a similar risk of intracranial hemorrhage, gastrointestinal bleeding and all major bleeding than those on antiplatelet agents (dotted line).

**Figure 2:**
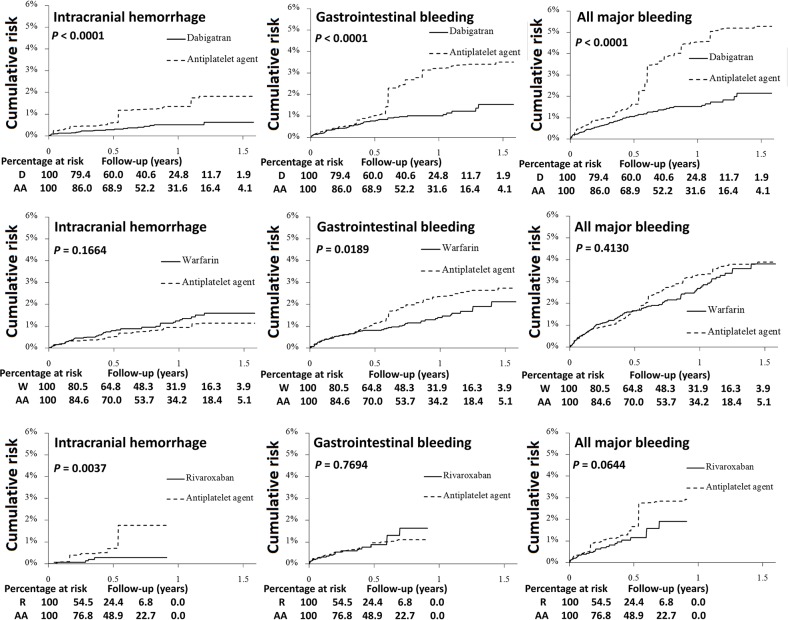
Cumulative risk of intracranial hemorrhage, gastrointestinal bleeding, and all major bleeding for NVAF patients treated with dabigatran, warfarin, or rixaroxaban versus AA after propensity score weighting Patients on dabigatran (solid line) had a lower risk of intracranial hemorrhage, gastrointestinal bleeding, and all major bleeding compared to those on antiplatelet agents (dotted line). Patients on warfarin (solid line) had a lower risk of gastrointestinal bleeding, and a similar risk of intracranial hemorrhage and all major bleeding compared to those on antiplatelet agents (dotted line). Patients on rivaroxabn (solid line) had a lower risk of intracranial hemorrhage, and a similar risk of gastrointestinal bleeding and all major bleeding compared to those on antiplatelet agents (dotted line).

The annual incidence of intracranial hemorrhage (ICH) was 0.6%, 1.3%, and 0.8%; the risk of gastrointestinal bleeding (GIB) was 1.3%, 1.5%, and 1.8%; and the risk of all major bleeding was 1.8%, 2.9%, and 2.6% for the dabiagtran, warfarin, and AA groups, respectively (Tables 4 and 5). Before weighting, dabigatran was associated with a significantly lower risk of GIB (Hazard ratio (HR): 0.68; *P* = 0.0200) and all major bleeding events (HR: 0.67; *P* = 0.0040) compared with AA. Warfarin carried a significantly higher risk of ICH compared to AA before adjustment (HR: 1.67; *P* = 0.0096). After weighting, dabigatran was associated with a significantly lower risk of ICH (HR: 0.36; *P*<0.0001), GIB (HR: 0.44; *P*<0.0001) and all major bleeding events (HR: 0.41; *P*<0.0001) compared with AA. The AA group was further categorized by use of aspirin and P_2_Y_12_ inhibitor (including clopidogrel and ticlopidine). Dabigatran was associated a lower risk of GIB and all major bleeding events compared with either aspirin or P_2_Y_12_ inhibitor (Table 4). Warfarin had a significantly lower risk of GIB (1.53 vs. 2.23%/year; HR: 0.68; *P*=0.0189) than AA after adjustment. The warfarin and AA groups had a similar risk of all major bleeding events (*P*=0.4130). It is noted that warfarin caused a significantly higher risk of ICH compared with P_2_Y_12_ inhibitor (HR: 3.35; *P*<0.0001) (Table 5). The annual incidence of ICH, GIB and all major bleeding was 0.5%, 2.0%, and 2.5% for rivaroxaban, respectively (Table 6). There was no significant difference in the risk of ICH, GIB or all major bleeding for rivaroxaban versus AA before adjustment, whereas rivaroxaban had a significantly lower risk of ICH compared with AA after adjustment (HR: 0.25; *P*=0.0037). It is noted that rivaroxaban caused a lower risk of all major bleeding compared with P_2_Y_12_ inhibitor (HR: 0.60; *P*=0.0336) (Table 6). Figures 1 and 2 show a clear separation of event curves for ICH, GIB, and all-major bleeding between the dabigatran and AA groups both before as well as after propensity score weighting adjustment.

**Table 4:** Incidence (per 100 person-years) of bleeding events in patients with non-valvular atrial fibrillation (AF) receiving dabigatran (D) and antiplatelet agents (AA)

	Intracranial hemorrhage				Gastrointestinal bleeding				All major bleeding			
	Crude events	Crude incidence	Adjusted^*^ events	Adjusted^*^ incidence	Crude events	Crude incidence	Adjusted^*^ events	Adjusted^*^ incidence	Crude events	Crude incidence	Adjusted^*^ events	Adjusted^*^ incidence
**(1) D versus AA**												
**D**	24	0.55	24	0.55	55	1.25	55	1.25	79	1.80	79	1.80
**(n=6,600)**		(0.33-0.77)		(0.33-0.77)		(0.92-1.58)		(0.92-1.58)		(1.40-2.20)		(1.40-2.20)
**AA**	50	0.76	82.08	1.47	115	1.76	159.32	2.86	167	2.55	242	4.34
**(n=8,238)**		(0.22-0.98)		(1.15-1.79)		(1.44-2.08)		(2.41-3.30)		(2.16-2.94)		(3.80-4.90)
**D vs. AAHR**		0.68		**0.36**		**0.68**		**0.44**		**0.67**		**0.41**
**(95% CI)**		(0.42-1.11)		**(0.23-0.57)**		**(0.50-0.94)**		**(0.32-0.59)**		**(0.52-0.88)**		**(0.32-0.53)**
***P value***		*0.1233*		***< 0.0001***		***0.0200***		***< 0.0001***		***0.0040***		***< 0.0001***
**(2) Aspirin**	46	0.77	97.19	1.72	89	1.49	176.44	3.13	137	2.29	274.60	4.87
**(n=7,516)**		(0.55-0.99)		(1.38-2.07)		(1.18-1.78)		(2.67-3.59)		(1.91-2.68)		(4.29-5.44)
**D vs. AspirinHR**		*0.68*		***0.31***		*0.80*		***0.40***		***0.75***		***0.37***
**(95% CI)**		*(0.41-1.11)*		***(0.20-0.49)***		*(0.57-1.12)*		***(0.30-0.54)***		***(0.57-0.99)***		***(0.29-0.47)***
***P value***		*0.1216*		***< 0.0001***		*0.2009*		***< 0.0001***		***0.0413***		***< 0.0001***
**(3) P_2_Y_12_ inhibitor**	9	1.08	26.20	0.49	31	3.73	130.37	2.45	40	4.82	156.57	2.95
**(n=1,057)**		(0.50-2.06)		(0.30-0.68)		(2.42-5.05)		(2.03-2.87)		(3.33-6.31)		(2.48-3.41)
**D vs. P_2_Y_12_ inhibitorHR**		*0.48*		*1.07*		***0.32***		***0.50***		***0.36***		***0.59***
**(95% CI)**		*(0.22-1.03)*		*(0.61-1.86)*		***(0.21-0.50)***		***(0.36-0.68)***		***(0.24-0.52)***		***(0.45-0.78)***
***P value***		*0.0601*		*0.8155*		***< 0.0001***		***< 0.0001***		***< 0.0001***		***0.0002***

CI = confidential interval; HR = hazard ratio

^*^ event numbers and incidence of antiplatelet agent versus dabigatran after propensity score weighting

**Table 5:** Incidence (per 100 person-years) of bleeding events in patients with non-valvular atrial fibrillation (AF) receiving warfarin (W) and antiplatelet agents (AA)

	Intracranial hemorrhage				Gastrointestinal bleeding				All major bleeding			
	Crude events	Crude incidence	Adjusted¶ events	Adjusted¶ incidence	Crude events	Crude incidence	Adjusted¶ events	Adjusted¶ incidence	Crude events	Crude incidence	Adjusted¶ events	Adjusted¶ incidence
**(1) W versus AA**												
**W**	51	1.30	51	1.30	60	1.53	60	1.53	115	2.93	115	2.93
**(n=5,338)**		(0.94-1.66)		(0.94-1.66)		(1.14-1.91)		(1.14-1.91)		(2.39-3.46)		(2.39-3.46)
**AA**	50	0.76	40.41	0.96	115	1.76	94.30	2.23	167	2.55	136	3.21
**(n=8,238)**		(0.22-0.98)		(0.66-1.25)		(1.44-2.08)		(1.78-2.68)		(2.16-2.94)		(2.68-3.76)
**W vs. AAHR**		**1.67**		1.34		0.86		**0.68**		1.13		0.90
**(95% CI)**		**(1.13-2.47)**		(0.89-2.02)		(0.63-1.17)		**(0.49-0.94)**		(0.89-1.44)		(0.70-1.16)
***P value***		***0.0096***		*0.1664*		*0.3384*		***0.0189***		*0.3054*		*0.4130*
**(2) Aspirin**	46	0.77	44.20	1.05	89	1.49	88.81	2.11	137	2.29	134.25	3.19
**(n=7,516)**		(0.55-0.99)		(0.74-1.36)		(1.18-1.80)		(1.67-2.55)		(1.91-2.68)		(2.65-3.73)
**W vs. AspirinHR**		***1.66***		*1.22*		*1.01*		***0.72***		*1.26*		*0.91*
**(95% CI)**		***(1.12-2.48)***		*(0.82-1.83)*		*(0.73-1.41)*		***(0.52-1.00)***		*(0.98-1.61)*		*(0.71-1.17)*
***P value***		***0.0126***		*0.3305*		*0.9394*		***0.0496***		*0.0678*		*0.4621*
**(3) P_2_Y_12_ inhibitor**	9	1.08	16.68	0.39	31	3.73	98.76	2.28	40	4.82	115.44	2.67
**(n=1,057)**		(0.50-2.06)		(0.20-0.57)		(2.42-5.05)		(1.83-2.73)		(3.33-6.31)		(2.18-3.15)
**W vs. P_2_Y_12_ inhibitorHR**		*1.18*		***3.35***		***0.40***		***0.66***		***0.60***		*1.09*
**(95% CI)**		*(0.58-2.40)*		***(1.92-5.82)***		***(0.26-0.62)***		***(0.48-0.92)***		***(0.42-0.86)***		*(0.84-1.41)*
***P value***		*0.6455*		***< 0.0001***		***< 0.0001***		***0.0125***		***0.0052***		*0.5167*

CI = confidential interval; HR = hazard ratio

^¶^ event numbers and incidence of antiplatelet agent versus warfarin after propensity score weighting

**Table 6:** Incidence (per 100 person-years) of bleeding events in patients with non-valvular atrial fibrillation (AF) receiving rivaroxaban (R) and antiplatelet agents (AA)

	Intracranial hemorrhage				Gastrointestinal bleeding				All major bleeding			
	Crude events	Crude incidence	Adjusted^*^ events	Adjusted^*^ incidence	Crude events	Crude incidence	Adjusted^*^ events	Adjusted^*^ incidence	Crude events	Crude incidence	Adjusted^*^ events	Adjusted^*^ incidence
**(1) R versus AA**												
**R**	5	0.48	5	0.48	21	2.03	21	2.03	26	2.51	26	2.51
**(n=3,167)**		(0.33-1.13)		(0.16-1.13)		(1.16-2.89)		(1.16-2.89)		(1.54-3.47)		(1.54-3.47)
**AA**	18	0.81	35.11	2.11	47	2.14	27.63	1.66	65	2.95	62.8	3.78
**(n=4,616)**		(0.44-1.20)		(1.42-2.81)		(1.52-2.75)		(1.04-2.81)		(2.24-3.67)		(2.85-4.72)
**R vs. AAHR**		0.52		**0.25**		0.90		1.09		0.80		0.65
**(95% CI)**		(0.20-1.47)		**(0.10-0.64)**		(0.54-1.51)		(0.61-1.93)		(0.51-1.27)		(0.41-1.03)
***P value***		*0.2306*		***0.0037***		*0.6930*		*0.7694*		*0.3398*		*0.0644*
**(2) Aspirin**	17	0.84	44.14	2.57	33	1.64	17.82	1.04	50	2.48	61.95	3.60
**(n=4,218)**		(0.44-1.24)		(1.81-3.32)		(1.08-2.20)		(0.55-1.52)		(1.79-3.17)		(2.70-4.50)
**R vs. AspirinHR**		*0.52*		***0.21***		*1.14*		*1.78*		*0.93*		*0.70*
**(95% CI)**		*(0.19-1.42)*		***(0.08-0.52)***		*(0.66-1.98)*		*(0.94-3.36)*		*(0.57-1.49)*		*(0.44-1.11)*
***P value***		*0.2008*		***0.0008***		*0.6488*		*0.0772*		*0.7503*		*0.1294*
**(3) P_2_Y_12_ inhibitor**	3	1.10	12.50	0.85	15	5.50	44.25	3.02	18	6.60	56.75	3.88
**(n=581)**		(0.23-3.21)		(0.38-1.33)		(2.72-8.28)		(2.13-3.91)		(3.55-9.65)		(2.87-4.88)
**R vs. P_2_Y_12_ inhibitorHR**		*0.41*		*0.52*		***0.37***		*0.63*		***0.38***		***0.60***
**(95% CI)**		*(0.10-1.72)*		*(0.18-1.47)*		***(0.19-0.74)***		*(0.37-1.06)*		***(0.21-0.70)***		***(0.38-0.96)***
***P value***		*0.2217*		*0.2161*		***0.0043***		*0.0803*		***0.0019***		***0.0336***

CI = confidential interval; HR = hazard ratio

^*^ event numbers and incidence of antiplatelet agent versus rivaroxaban after propensity score weighting

### Predictors of major bleeding

Multivariable analysis showed that age ≥ 75 years was statistically significant independent predictor of major bleeding in the dabigatran, warfarin as well as AA groups (*P*<0.01 in all groups). Presence of heart failure (HR, 1.85; *P*=0.0195) and chronic kidney disease (HR, 2.28; *P*=0.0005) were the other independent predictors of major bleeding events in the dabigatran group. Additionally, the presence of stroke/TIA and use of non-steroid anti-inflammatory drugs were significant independent predictors of major bleeding in the AA and warfarin groups (*P*<0.05 in both groups). Female and use of non-steroid anti-inflammatory drugs were significant independent predictors of major bleeding in the rivaroxaban group (Table 7).

**Table 7:** Multivariable analysis of predictors of all major bleeding in 4 treatment groups

	Dabigatran (n=6,600)Hazard ratio (95%CI); *P*-value	Rivaroxaban (n=3,167)Hazard ratio (95%CI); *P*-value	Warfarin (n=5,338)Hazard ratio (95%CI); *P*-value	Antiplatelet agent (n=8,238)Hazard ratio (95%CI); *P*-value
**Age >=75**	**2.13(1.27-3.59); 0.0043**	2.15(0.85-5.45); 0.1056	**2.89(1.90-4.39); <0.0001**	**3.09(2.19-4.34); <0.0001**
**Male**	1.14(0.72-1.80); 0.5739	**0.41(0.18-0.97); 0.0421**	1.00(0.69-1.45); 0.9877	1.61(0.85-1.59); 0.3493
**Stroke/TIA**	1.43(0.92-2.24); 0.1148	1.37(0.61-3.05); 0.8769	**1.51(1.01-2.24); 0.0443**	**1.82(1.17-2.82); 0.0079**
**Hypertension**	1.57(0.67-3.66); 0.3009	0.91(0.26-3.16); 0.8769	1.22(0.73-2.05); 0.4445	**1.47(1.04-2.10); 0.0315**
**Congestive heart failure**	**1.85(1.10-3.09); 0.0195**	1.22(0.46-3.28); 0.6881	1.47(0.92-2.35); 0.1054	1.04(0.59-1.80); 0.9031
**Diabetes mellitus**	0.68(0.43-1.10); 0.1150	0.89(0.39-2.02); 0.7789	0.90(0.60-1.36); 0.6168	1.05(0.73-1.51); 0.7877
**Malignancy**	1.14(0.55-2.37); 0.7311	0.46(0.06-3.38); 0.4418	1.34(0.76-2.43); 0.3025	1.42(0.88-2.27); 0.1478
**Chronic kidney disease**	**2.28(1.43-3.64); 0.0005**	1.06(0.41-2.73); 0.9016	1.39(0.90-2.13); 0.1346	**1.77(1.18-2.66); 0.0062**
**Chronic liver disease**	1.07(0.66-1.75); 0.7809	0.98(0.40-2.42); 0.9682	0.75(0.46-1.22); 0.2452	0.80(0.48-1.34); 0.3966
**Use of NSAIDs**	1.51(0.94-2.42); 0.0879	**2.31(1.04-5.16); 0.0410**	**1.78(1.22-2.60); 0.0026**	**1.75(1.28-2.39); 0.0005**

The abbreviations as in Table 1

### Bleeding risk in selected subgroups

**Figure 3:**
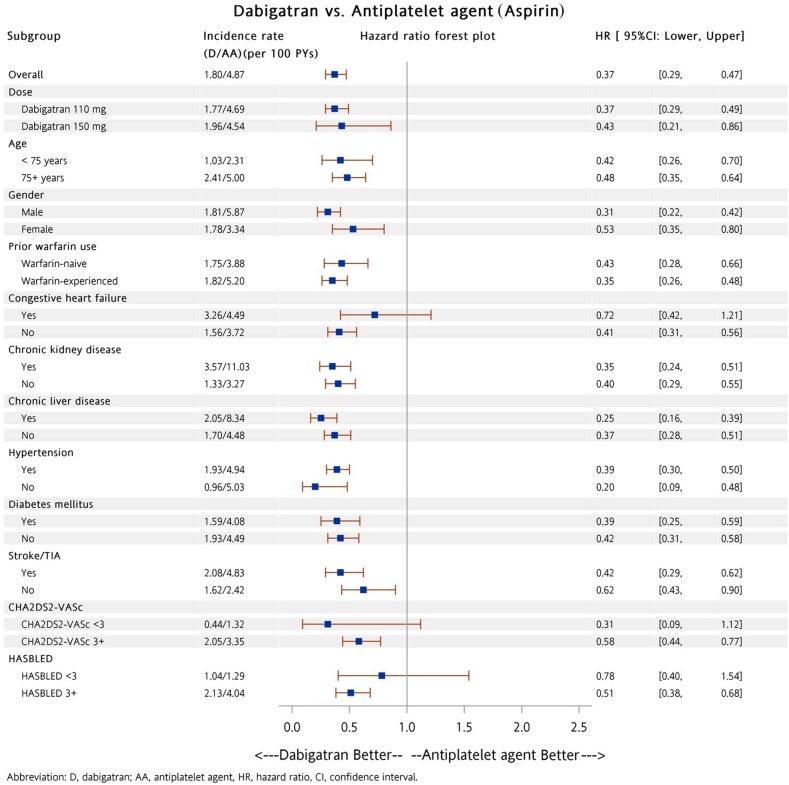
Forest plot of hazard ratio of all major bleeding for NVAF patients taking dabigatran versus aspirin after propensity score weighting Dabigatran was associated with a lower risk of all major bleeding events compared with aspirin in most subgroups. The abbreviations as in Figure 1.

**Figure 4:**
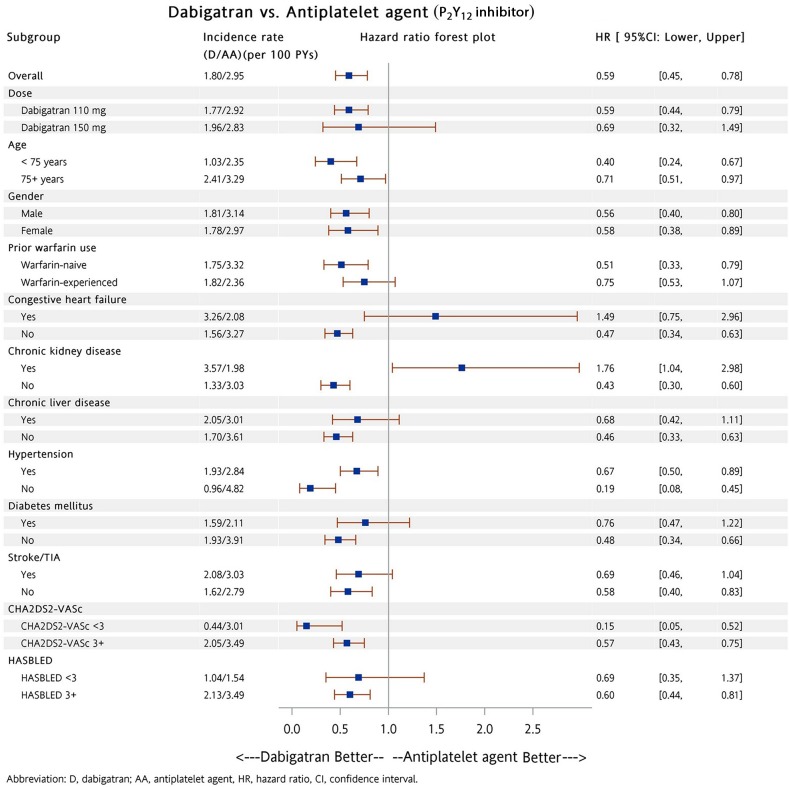
Forest plot of hazard ratio of all major bleeding for NVAF patients taking dabigatran versus P_2_Y_12_ inhibitor after propensity score weighting Dabigatran was associated with a lower risk of all major bleeding events compared with P_2_Y_12_ inhibitor in most subgroups. The abbreviations as in Figure 1.

**Figure 5:**
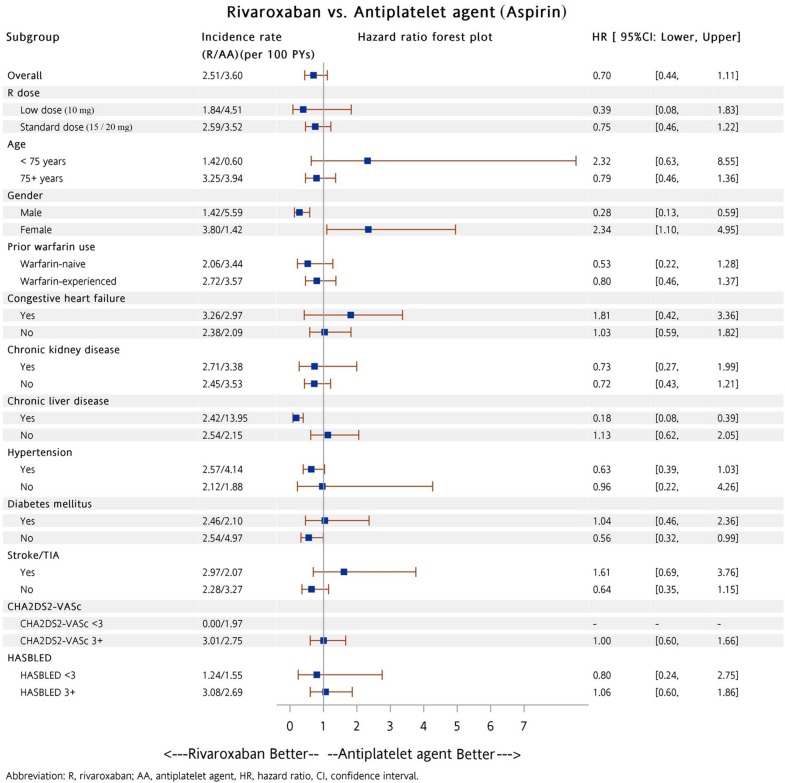
Forest plot of hazard ratio of all major bleeding for NVAF patients taking rivaroxaban versus aspirin after propensity score weighting Rivaroxaban was associated with a similar risk of all major bleeding as compared with aspirin users in most subgroups. The abbreviations as in Figure 1.

**Figure 6:**
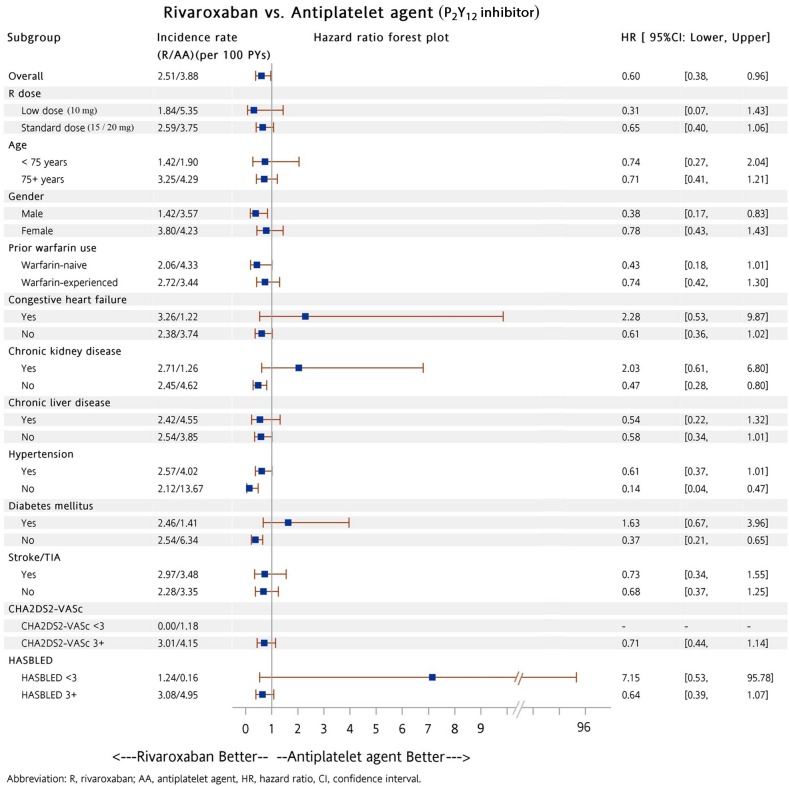
Forest plot of hazard ratios of all major bleeding for NVAF patients taking rivaroxaban versus P_2_Y_12_ inhibitor after propensity score weighting Warfarin was associated with a similar risk of all major bleeding as compared with P_2_Y_12_ inhibitor in most subgroups. The abbreviations as in Figure 1.

**Figure 7:**
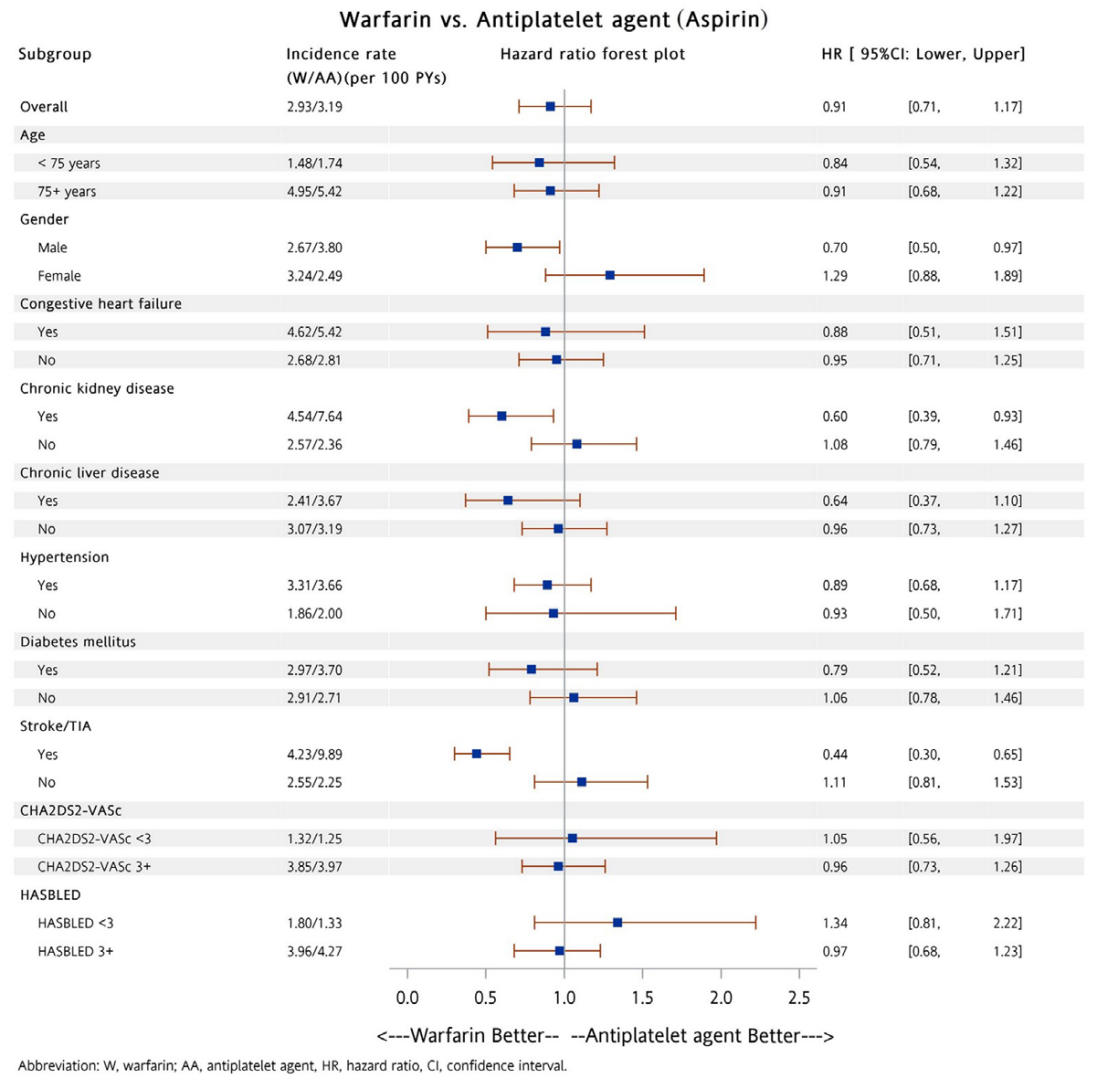
Forest plot of hazard ratios of all major bleeding for NVAF patients taking warfarin versus aspirin after propensity score weighting Warfarin was associated with a similar risk of all major bleeding as compared with aspirin in most subgroups. The abbreviations as in Figure 1.

**Figure 8:**
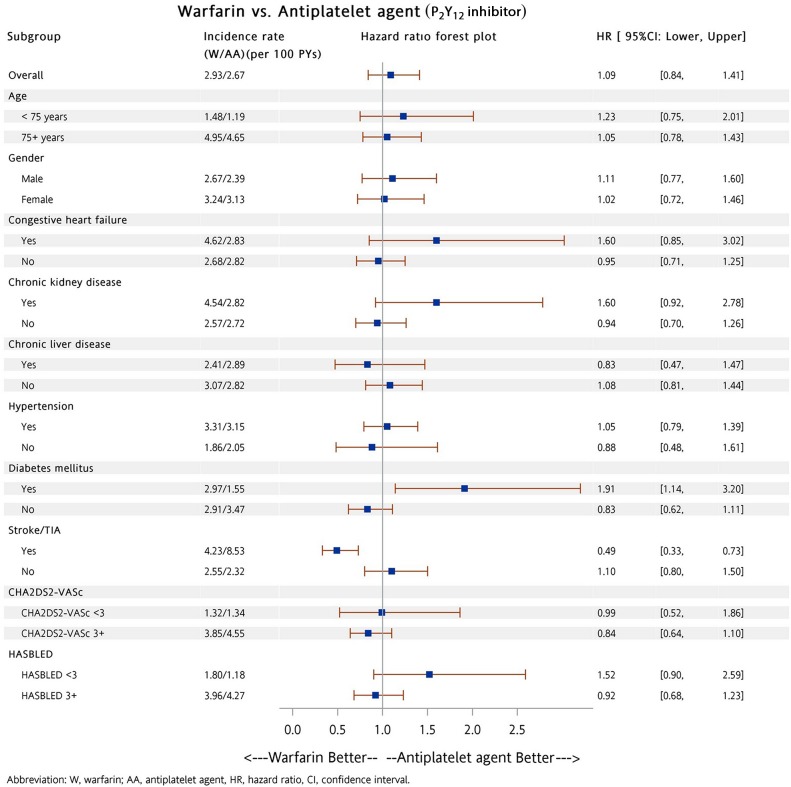
Forest plot of hazard ratios of all major bleeding for NVAF patients taking warfarin versus P_2_Y_12_ inhibitor after propensity score weighting Warfarin was associated with a similar risk of all major bleeding as compared with P_2_Y_12_ inhibitor in most subgroups. The abbreviations as in Figure 1.

Subgroup analysis was performed to determine whether patients in the dabigatran and warfarin groups had a lower risk of all major bleeding compared to patients taking either aspirin or P_2_Y_12_ inhibitor (including clopidogrel and ticlopidine). A total of 5,822 (88%) and 164 (5%) patients were prescribed low dose dabigatran (110 mg twice daily) and rivaroxaban (10 mg once daily), respectively. Among these patients, there were 3,846 (58%) and 2,046 (65%) patients with a previous history of warfarin exposure, respectively. Our analysis showed patients on dabigatran had a lower risk of major bleeding compared to patients with either aspirin (Figure 3) or P_2_Y_12_ inhibitor (Figure 4) in most subgroups. In contrast to patients in the dabigatran group, patients in the rivaroxaban and warfarin groups had a similar risk of major bleeding compared to either aspirin or P_2_Y_12_ inhibitor group in most subgroups (Figures 5 to 8).

## DISCUSSION

This large population-based study investigated the safety profiles of dabigatran, rivaroxaban, and warfarin versus AA with a specific focus on Asians with NVAF taking antithrombotic agents. Our data showed that nearly 90% of patients taking low doses of dabigatran (110 mg twice daily) had a significantly lower risk of ICH, GIB and all major bleeding compared to patients taking AA after adjustment. Importantly, patients in the warfarin and AA groups had a similar risk of ICH and all major bleeding after adjustment. Approximately 87% of patients taking low doses of rivaroxaban (10 or 15 mg once daily) had a significantly lower risk of ICH, but a similar risk of GIB and all major bleeding compared to patients in the AA group after adjustment. Subgroup analysis confirmed that dabigatran was associated with a lower risk of major bleeding compared with AA in most subgroups, whereas warfarin and rivaroxaban were associated with a similar risk of major bleeding compared with AA in most subgroups.

Aspirin has been shown to be ineffective for stroke prevention among whites with AF. [1, 13] Recent data from a Japanese trial and a Hong Kong cohort also indicated that aspirin was not effective for stroke prevention among Asians with AF. [14, 15] It is estimated that as high as 25% of AF patients take aspirin for stroke prevention in Asia, possibly due to an impression that aspirin is safer than warfarin. [16] There are several explanations for the unique phenomenon of warfarin underutilization in Asia: It has been reported a 2 to 4-fold higher risk of ICH in Asians treated with warfarin compared with whites over the entire therapeutic range. [4, 5] It seemed that it is more difficult to maintain the therapeutic range of international normalized ratio (INR) of 2 to 3 among Asians on warfarin compared with non-Asians, possibly due to ethnicity and drug interaction. [6, 17] For the above reasons, physicians were relatively reluctant to prescribe warfarin in Asia.

The BAFTA and recent Hong Kong cohort studies indicated there was no difference in the risk of cerebrovascular hemorrhage between warfarin and aspirin. [13, 15, 23] The 2016 ESC guidelines for AF reported that the risk of major bleeding or ICH associated with aspirin is not significantly different from that of oral anticoagulants, which was mainly based on results from the BAFTA study. [19] In the contrast, a recent meta-analysis which pooled several randomized trials investigating the major bleeding risk associated with aspirin versus vitamin K antagonists (VKA), including the recently published WARCEF trial, indicated that the risk of major bleeding was substantially higher for VKA targeting the current usual INR range compared to aspirin. [20] Our study showed that the annual risk of ICH in our Asian cohort taking warfarin was 1.3%, which was compatible to the reported risk of 1.1 to 2.5% in the Asian subgroup analyses from the four pivotal NOACs trials. [21, 22] Although the risk of ICH in the warfarin group was significantly higher compared to the AA group (0.76%) before adjustment, warfarin did not cause a higher ICH risk than AA after co-morbidity adjustment. Additionally, our study indicated that AA use was indeed associated with a higher risk of GIB compared with warfarin. Considering the similar incidence of overall major bleeding events between the warfarin and AA groups either before or after adjustment, our study concluded that AA was not a “safer” alternative to warfarin in reducing major bleeding events in Asians.

There are few studies which directly compared the bleeding risk between NOACs and aspirin. Until now, only the AVERROES trial reported that apixaban was superior to aspirin for stroke prevention in AF patients who were deemed unsuitable for warfarin treatment, and apixaban reduced the risk of stroke/systemic embolism from 3.7%/year to 1.6%/year compared with aspirin. [7] There was no significant difference in major bleeding risk between apixaban and aspirin (1.4%/year versus 1.2%/year). Ho et al. investigated the ICH risk in a real-world cohort of Chinese AF patients receiving warfarin, dabigatran, aspirin, or no therapy. [17, 18] The incidence of ICH was lowest in patients on dabigatran (0.32%/year) as compared with those on aspirin (0.80%/year). However, the patient numbers in this study were very limited, with only 393 and 3,600 patients taking dabigatran and aspirin, respectively. In contrast, our study is the largest ever examination of the safety of anti-thrombotic therapy with an enrollment of 6,600 and 8,238 Asians taking dabigatran and AA respectively. Our data indicated that dabigatran was significantly associated with a lower risk of all major bleeding compared with AA during identical follow-up periods. The RELY subgroup analysis previously indicated that the major bleeding risk was significantly lower among Asians on either standard or low dose dabigatran compared to warfarin. [24] In contrast, the ROCKET-AF trial and recent real world data showed that rivaroxaban, which was in contrast to other NOACs (apixaban, dabigatran, and edoxaban), has a similar risk of major bleeding comparable with warfarin. [25–30] The recent real world data further demonstrated that rivaroxaban caused more ICH, GIB and all major bleeding than dabigatran. [31] It was therefore expected that rivaroxaban may be associated with a comparable risk of major bleeding as AA. However, our present data showed that in real world practice in Asia, both dabigatran and rivaroxaban were associated with a lower risk of ICH compared to AA.

### Study limitations

First, we cannot evaluate several laboratory parameters including hemoglobin, labile INR, renal and liver function test, and other potential confounders from the national database. Second, although we included an extensive number of variables in our model and achieved a close balance for most factors, residual confounding by unmeasured factors cannot be excluded. Third, this study had a relative short follow-up period. Fourth, the data regarding apixaban and edoxaban were not available. Finally, because dabigatran largely depends on renal excretion, it is possible that the physicians could avoid the use of dabigatran for patients with a further impaired renal function. Consequently, the baseline renal function may be better for the dabigatran group compared to other group, which may explain for the better safety profiles with dabigatran compared to other study group. Because the NHIRD does not have important laboratory data including serum creatinine level, we cannot clarify the issue if the favorable outcome of safety issue for dabigatran was contributed from the better baseline renal function or not.

## MATERIALS AND METHODS

### Study population

The Taiwan National Health Insurance (NHI) system is a mandatory universal health insurance program providing comprehensive medical care coverage to all Taiwanese residents. The NHI research database (NHIRD) of the National Health Research Institutes of Taiwan included detailed health care information for more than 99% of the Taiwanese population (23 million enrollees) in 2014. [8] The study was approved by the Institutional Review Board of Chang-Gung Medical Foundation.

**Figure 9:**
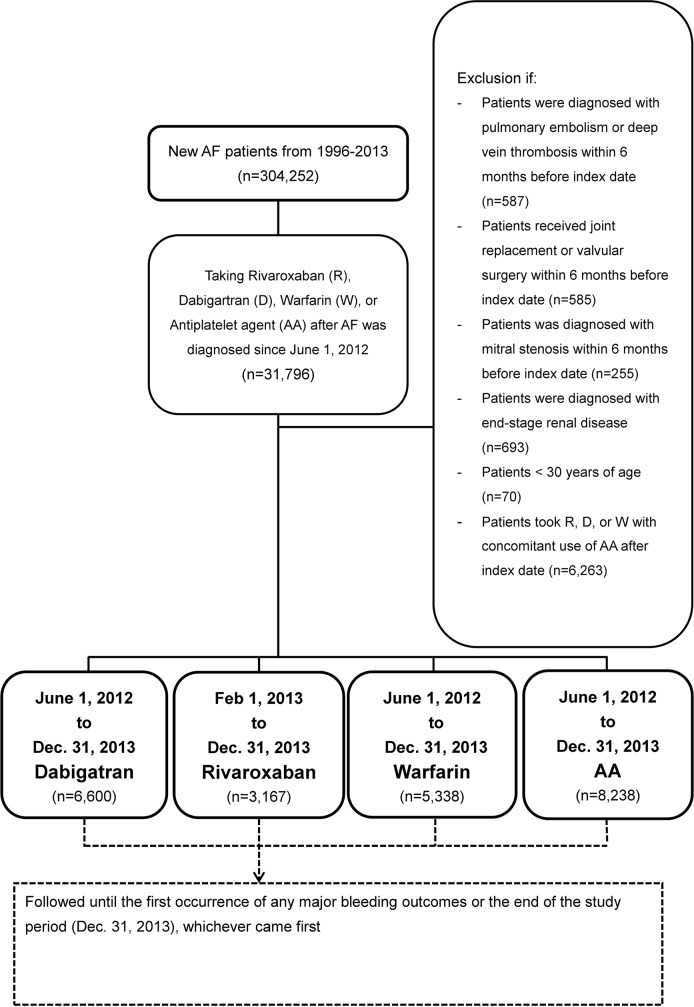
Enrollment of patients with NVAF A total of 304,252 new non-valvular atrial fibrillation (NVAF) patients including 6,600 dabigatran (D), 3,167 rivaroxaban (R), 5,338 warfarin (W), and 8,238 antiplatelet agent (AA) users were enrolled in this study from June 1, 2012 to December 31, 2013. Rivaroxaban was enrolled in this study from February 1, 2013 duo to its approval at that day in Taiwan.

This study evaluated a national cohort with four study groups (dabigatran, rivaroxaban, warfarin and AA). Figure 9 showed the flowchart of the study cohort. A total of 304,252 new AF patients were identified between January, 1996 and December, 2013. AF was diagnosed based on *International Classification of Diseases 9th, Clinical Modification (ICD-9-CM)* codes (427.31), in either in-patient once or outpatient department twice. We identified patients with a first time prescription of NOACs dabigatran (from June 1, 2012) and rivaroxaban (from February 1, 2013), as well as patients who started warfarin or AA treatment (from June 1, 2012) up to December 31, 2013 after an established diagnosis of AF. [9, 32] Index date for each study group was defined as the first date when medication was prescribed. The excluded patients were presented in Figure 9. Patients taking NOACs or warfarin with concomitant use of AA after the index date (n=6,263) were also excluded. The follow-up period was from the index date until the occurrence of the first study outcomes or the end of the study period (December 31, 2013), whichever came first.

### Study outcomes

The three study outcomes defined to determine the safety profiles for dabigatran, rivaroxaban, warfarin and AA were 1) ICH, 2) GIB, and 3) all major bleeding events. The study outcomes had to be a discharge diagnosis. Major bleeding was defined a hospitalized bleeding event with hemorrhage into a critical site (e.g., gastrointestinal, intracranial, intraspinal, intraarticular, intraocular, pericardial, retroperitoneal, or intramuscular with compartment syndrome). ICH was defined with the use of codes for atraumatic hemorrhage. [9] The ICD-9-CM codes used in the study outcomes are summarized in Table 8. It notes that the same patient could have more than one study outcomes and could have the same study outcomes several times during the study duration. However, only the study outcomes which appeared first were counted, because patients were managed differently based on their study outcomes.

**Table 8:** International Classification of Disease (9^th^ edition) Clinical Modification (ICD 9-CM) codes used to define the co-morbidities and clinical outcome in the study cohort

Disease	ICD-9 Codes	Diagnosis definition
Atrial fibrillation	427.31	Discharge or outpatient department ≥2
Ischemic stroke	433, 434, 436	Discharge
Transient ischemic attack	435	Discharge
Peripheral arterial occlusive disease	440.2	Discharge
Myocardial infarction	410, 411, 412	Discharge
Congestive heart failure	428	Discharge
Hypertension	401, 402	Outpatient department ≥2
Diabetes mellitus	250	Outpatient department ≥2
Hyperlipidemia	272	Outpatient department ≥2
Chronic gout	274.0, 274.10, 274.11, 274.19, 274.81, 274.82, 274.89, 274.9	Outpatient department ≥2
Chronic lung disease	490, 491.0, 491.1, 491.20-491.22, 491.8, 491.9, 492.0, 492.8, 493.00-493.02 493.10-493.12, 493.20-493.22, 493.81, 493.82, 493.90-493.92, 494.0, 494.1, 495.8, 495.9, 496, 500, 502, 503, 504, 505, A323, A325	Outpatient department ≥2
Chronic kidney disease	580-589	Outpatient department ≥2
Chronic liver disease	570, 571, 572	Outpatient department ≥2
Malignancy	140.0-208.9	Outpatient department ≥2
Intracranial hemorrhage	430, 431, 432, 852, 853	Discharge
Gastrointestinal bleeding	456.0, 456.2, 455.2, 455.5, 455.8, 530.7, 530.82, 531.0-531.6, 532.0-532.6, 533.0-533.6, 534.0-534.6, 535.0-535.6 537.83, 562.02, 562.03, 562.12 562.13 568.81, 569.3, 569.85, 578.0, 578.1, 578.9	Discharge
Other critical site bleeding	423,0, 459.0, 568.81, 593.81, 599.7, 623.8, 626.32, 626.6, 719.1, 784.7, 784.8, 786.3	Discharge

### Covariates

Information for risk factors of cardiovascular events and bleeding, and use of medication at baseline was obtained from claim records with the above diagnoses or medication codes prior to the index date. History of bleeding was confined to events within 6 months preceding the index date. A history of specific prescribed medicines was confined to medications used at least once within 3 months preceding the index date. The CHA_2_DS_2_-VASc score predicted the risk of ischemic stroke in AF patients, and the HAS-BLED score predicted the risk of bleeding in AF patients treated with oral anticoagulant or antiplatelet. [10, 11]

### Statistical analysis

The effects of dabigatran, rivaroxaban and warfarin on study outcomes were estimated using the propensity score method. Inverse probability of treatment weights (IPTW) of propensity scores was used to balance covariates across the two study groups. [12] The balance of potential confounders at baseline (index date) between the two study groups was assessed using absolute standardized mean difference (ASMD), rather than using statistical testing, because balance is a property of the sample and not of an underlying population. The value of ASMD ≤0.1 indicates a negligible difference in potential confounders between the two study groups. Risk of study outcomes over time for dabigatran, rivaroxaban or warfarin compared with AA (reference) was obtained using survival analysis (Kaplan-Meier method for univariate analysis and Cox proportional hazards regression for multivariate analysis) after IPTW. Statistical significance was defined as a *P* value of <0.05. All statistical analyses were performed using SAS 9.3 (SAS Institute Inc., Cary, North Carolina).

## CONCLUSIONS

Dabigatran use was associated with significantly lower risk of all major bleeding events compared to AA in a large Asian cohort with NVAF. In contrast, patients in rivaroxaban groups had a similar risk of major bleeding compared to the AA group. The findings implicated that dabigatran may be a safer alternative to aspirin for Asian AF patients.

## Abbreviations

AF: atrial fibrillation
GIB: gastrointestinal bleeding
ICH: intracranial hemorrhage
INR: international normalized ratio
NOAC: non-vitamin K antagonist oral anticoagulant
NVAF: non-valvular atrial fibrillation
OAC: oral anticoagulant
VKA: vitamin K Antagonists
NHIRD: National Health Insurance Research Database
TTR: time in therapeutic range
